# Boosting SARS-CoV-2 detection combining pooling and multiplex strategies

**DOI:** 10.1038/s41598-022-12747-8

**Published:** 2022-05-23

**Authors:** Isadora Alonso Correa, Tamires de Souza Rodrigues, Alex Queiroz, Leon de França Nascimento, Thiago Wolff, Rubens Nobumoto Akamine, Sergio Noboru Kuriyama, Luciana Jesus da Costa, Antonio Augusto Fidalgo-Neto

**Affiliations:** 1 Departamento de Virologia, Instituto de Microbiologia Paulo de Góes, Universidade Federal do Rio de Janeiro, Rio de Janeiro, 21941-617 Brazil; 5 Laboratório de Genética e Imunologia das Infecções Virais, Departamento de Virologia, Instituto de Microbiologia Paulo de Góes, Universidade Federal do Rio de Janeiro, Avenida Carlos Chagas Filho, 373, CCS, Bloco I, Lab.I-SS 048, Cidade Universitária, Rio de Janeiro, RJ 21941-902 Brazil; 2 Instituto Senai de Inovação em Química Verde, Rua Moraes e Silva, 53-9, Maracanã, Rio de Janeiro, RJ 20271-030 Brazil; 3 Centro de Inovação SESI em Saúde Ocupacional, Rio de Janeiro, 20271-030 Brazil; 4 Centro de Pesquisa Leopoldo Américo Miguez de Mello (CENPES)-Petrobrás, Rio de Janeiro, 21941-915 Brazil

**Keywords:** Microbiology, Virology, SARS-CoV-2

## Abstract

RT-qPCR is the gold standard technique available for SARS-CoV-2 detection. However, the long test run time and costs associated with this type of molecular testing are a challenge in a pandemic scenario. Due to high testing demand, especially for monitoring highly vaccinated populations facing the emergence of new SARS-CoV-2 variants, strategies that allow the increase in testing capacity and cost savings are needed. We evaluated a RT-qPCR pooling strategy either as a simplex and multiplex assay, as well as performed *in-silico* statistical modeling analysis validated with specimen samples obtained from a mass testing program of Industry Federation of the State of Rio de Janeiro (Brazil). Although the sensitivity reduction in samples pooled with 32 individuals in a simplex assay was observed, the high-test sensitivity was maintained even when 16 and 8 samples were pooled. This data was validated with the results obtained in our mass testing program with a cost saving of 51.5% already considering the expenditures with pool sampling that were analyzed individually. We also demonstrated that the pooling approach using 4 or 8 samples tested with a triplex combination in RT-qPCR is feasible to be applied without sensitivity loss, mainly combining Nucleocapsid (N) and Envelope (E) gene targets. Our data shows that the combination of pooling in a RT-qPCR multiplex assay could strongly contribute to mass testing programs with high-cost savings and low-reagent consumption while maintaining test sensitivity. In addition, the test capacity is predicted to be considerably increased which is fundamental for the control of the virus spread in the actual pandemic scenario.

## Introduction

Since the first reported cases in Wuhan (China) in December 2019, COVID-19 (Coronavirus Disease 19) has spread around the world, and in March 2020 was classified by the World Health Organization (WHO) as a pandemic^1^. By November 30, 2021, the number of people infected by Severe Acute Respiratory Syndrome Coronavirus 2 (SARS-CoV-2) exceeded more than 300 million worldwide, with more than five million accumulated deaths related to COVID-19 heavily affecting the global economy and health systems^2^. Within less than a year from the beginning of the pandemic, several countries started vaccination campaigns using one of the 13 available vaccines around the world. However, vaccinations rates are unequal, with countries like Portugal, United Arab Emirates and Chile reaching more than 80% of its population fully vaccinated while others have under 40% of its population totally immunized such as Nigeria (2.4%), Russia (36.2%) and South Africa (23.8%)^3^. Besides the differences in vaccine access, the length of immune response after vaccination and the emergence of newe viral variants are factors that could impair vaccine efficacy leading to SARS-CoV-2 local outbreaks with a high risk of global dissemination.

Pharmaceutical treatment options are still limited and measures that maintain viral vigilance, such contact tracing and quickly diagnostic and isolation of confirmed cases, remain key factors to control the spread of SARS-CoV-2^4^. In this context, testing is essential for transmission control and the maintenance of normal activities after lockdown especially if considering the asymptomatic cases that usually are not tested in the standard public health surveillance.

Mass testing is a critical strategic approach for epidemic control, pushing the global demand for diagnosis tests exceeding the supply capacity^5^. The current wave of Omicron variant exemplifies the problem of test access and availability. Several regions are facing shortage of SARS-CoV-2 diagnostic tests, which will negatively impact on the viral spread control since isolation of positive cases will be less efficient. Mid and low-income countries have always tested at lower rates when compared to high income countries, for instance, up to October 22, 2021, the United Kingdom present a total of 8,641,225 cumulative cases and performed, until the 11th day, 4,558,362 diagnostic tests per million inhabitants while United States and Brazil with 44,940,696 and 21,680,488 cumulative cases, respectively, tested 1,971,408 and 297,348 per million inhabitants^2,6^.

Virus detection in human respiratory tract samples is the standard diagnosis of ongoing acute infections, which represents a reliable approach to manage virus transmission^7^. WHO recommends quantitative Reverse-Transcription Polymerase Chain Reaction (RT-qPCR) as the standard method for molecular SARS-CoV-2 detection due to its high sensibility and specificity^8^. Frequently, the protocols used for SARS-CoV-2 diagnosis includes the detection of two viral targets and one human internal control performed either in single reactions or in a multiplex format to each patient tested to increase the reliability of the results^8,9^.

Pooling methodologies are based on the combination of multiple specimens in single sample analysis^10,11^ and could be applied for screening large numbers of individuals during diagnosis routine, being less expensive and time-consuming than individual testing^12^. Another strategy that could be used for diagnostic optimization is the multiplex assay, which aims to reduce the RT-qPCR reactions needed performing one reaction for both viral targets and the human internal control.

Furthermore, both methodologies (pooling test and multiplex assay) could help more countries to perform mass testing; however, the combination of both strategies has not been fully explored, even though the cost savings can be truly reached. The loss of sensitivity caused by dilution of samples, the choice of a combination of targets and probes for multiplex assay and the increase of false-negative results are some problems that need to be considered if both methodologies would be employed.

In this work, we describe a detailed optimization of pooling test protocol and a combination of pooling and multiplex assay to offer an economically viable approach for reliable massive diagnosis services according to the prevalence of positive cases on the evaluated population.

## Results

### Evaluation of pooling samples with isolated SARS-CoV-2

Serial dilution curves of a SARS-CoV-2 viral stock showed a low detection limit for both N1 and N2 targets (0.001 and 0.01 infectious viral particles/mL, respectively) (Fig. S1A). We measured total viral genomic RNA in the titrated viral stock and calculated a total of 10^3^ less infectious particles than the total viral particles present, thus the detection limit of this test is 1 RNA copy. Mixtures of SARS-CoV-2 titrated stocks and SARS-CoV-2 negative VTM pools in different proportions (1:0; 1:4; 1:8; 1:12; 1:16) were tested. Although C_t_ values increased when viral stocks were diluted in VTM, when compared to the undiluted virus, the accuracy in detecting SARS-CoV-2 targets was maintained. At least 10 infectious virus particles were still detected with pooled viral stocks (Fig. S1B,C).

### Evaluation of pooling samples with clinical samples

**Figure 1:**
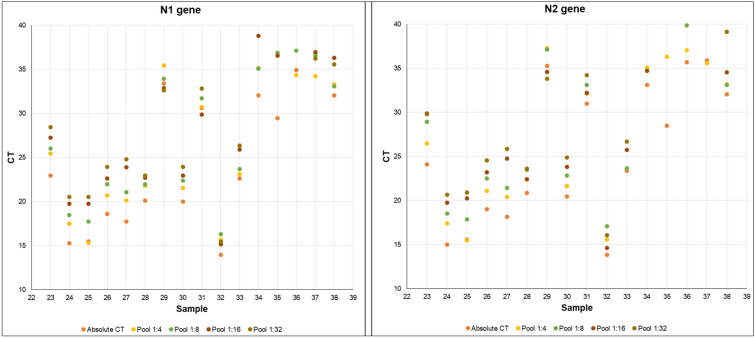
Pooling test examples of samples with distinct CT ranges. The values from all samples tested are presented in Table S1. 1:4, 1:8, 1:16 and 1:32 correspond to one positive sample pooled with other 3, 7, 15 and 32 negative samples, respectively.

Pooling strategy with up to 32 samples in a single pool showed that SARS-CoV-2 detection, considering the N1 gene, was highly sensitive in samples with individual C_t_ values below 29, with all tested samples being positive according to the CDC criteria (Fig. 1)^7^. However, for lower viral loads, samples with individual C_t_ higher than 29, generally observed in patients at the beginning and the end of the infection process, poor results were found for pools consisting of 16 or 32 specimens. Detection of the N1 gene was possible in 86% of samples for the 1:16 pool and 57% of samples for the 1:32 pool (Fig. 1A). The sensitivity of the test also decreased for the N2 gene, with detection possible in 86% of samples for the 1:8 pool, 57% for the 1:16 pool, and 43% for the 1:32 pool (Fig. 1B). It was observed that samples with C_t_ values higher than 34 reduced the test sensitivity when 16 or 32 patients were pooled (Fig. 1). Absolute C_t_ values for all the sample pools and RNA pools tested are summarized in Tables S1 and S2, respectively.

PCA analysis performed with pooled VTM samples and pooled RNA samples did not detect any group pattern amongst them (Fig. S2; PC-1 = 98% and PC-2 = 1%; Tables S1 and S2), which means that no difference was observed between both pooling approaches. Negative and positive controls, as well as the C_t_ curve from pools using only negative patient samples, were validated in our analyses (Fig. S3).

### In silico pooling analyses

To assess the advantages of the pooling approach, we used previous RT-qPCR results obtained in the diagnostic analyses performed with industrial workers of Rio de Janeiro state as a base to calculate the prevalence rates (%) of positive cases and to build the statistical modeling methodology. According to the in-silico methodology established, it was possible to construct a matrix evaluating the cost-savings for each pool size given positive cases prevalence. Based on this matrix, it is possible to suggest ideal pool sizes according to the prevalence rates and the cost-saving percentages on any given population (Table 1).

**Table 1:** Matrix representing pool size recommendation according to the pool size and prevalence rate of positive cases. Matrix values represent the predicted cost savings for each condition.

Pool size	Prevalence rate of positive cases (%)					
	1	2.5	5	7.5	10	15
2	48.01	45.06	40.25	35.56	31.00	22.25
4	71.06	65.37	56.45	48.21	40.61	27.20
8	79.77	69.17	53.84	41.10	30.55	14.75
12	80.31	65.47	45.70	30.90	19.91	5.89
16	78.90	60.44	37.76	22.48	12.28	1.18
20	76.79	55.27	30.85	16.03	7.16	− 1.12
24	74.40	50.30	25.03	11.23	3.81	− 2.14
28	71.90	45.65	20.21	7.70	1.66	− 2.52
32	69.37	41.35	16.25	5.13	0.31	− 2.57

**Figure 2:**
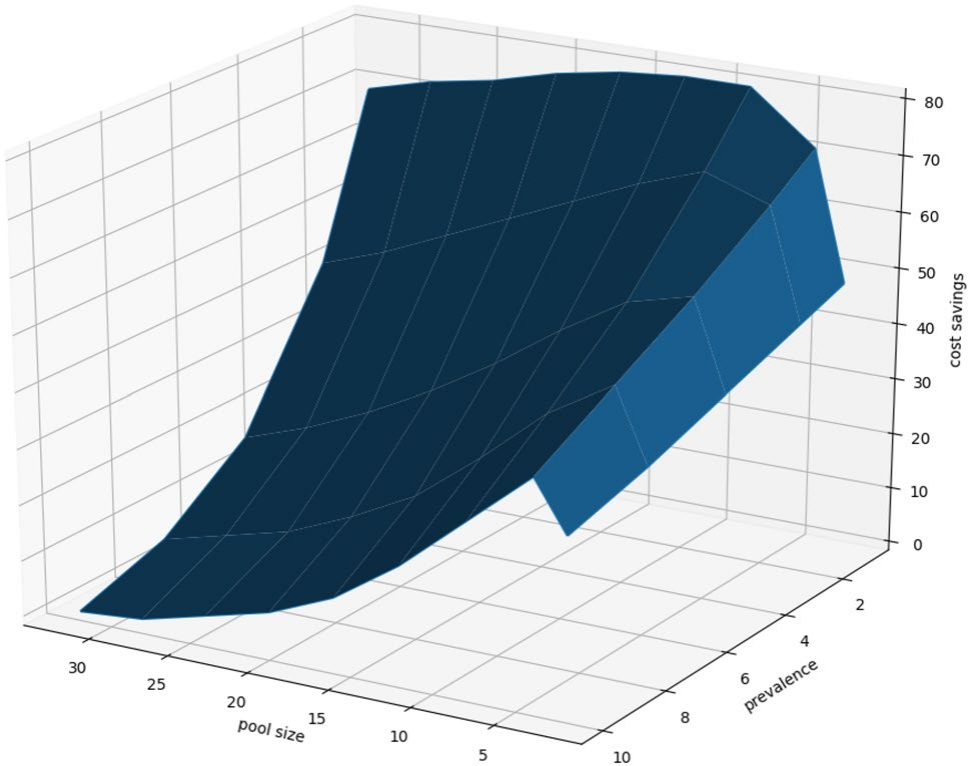
Pooling test savings given pool size and prevalence. Cost savings surface depicting optimal savings crests at pool sizes around 4 and 8. For populations with prevalence around 1%, many pool sizes are profitable, but as prevalence increases, costs savings are drastically reduced.

This mathematical modeling shows that populations with prevalence rates as low as 1% may reduce costs up to 80% using up to 8 or 12 specimens per pool. However, as the prevalence rate increases, the cost saving is drastically reduced in pools with a large number of samples. The need to process single analysis from the pool to identify the positive individuals increases the overall cost. Considering prevalence values equal to 5, 7.5, and 10%, the best results were observed for 1:4 pooling, with an economy of 57, 48, and 41%. As such, for prevalence rates higher than 1% but lower than 10%, pooling sizes of 4 and 8 return better cost savings in comparison to larger pool sizes. The cost modeling also demonstrates that as both pool sizes and population positive prevalence increases, the savings become marginally lower until they surpass the value of a single test, limiting the optimal cost savings to a well-defined bounded range (Fig. 2).

### Validation of pooling strategies with clinical samples

Previous data of the COVID-19 diagnostic performed with industrial workers showed a prevalence of 7.8% positive cases in the evaluated population. According to the in-silico analysis performed, pooling using four samples was the best choice for cost optimization (Table 1). To assess the precision of the in-silico model, the mass testing program for industrial workers of Rio de Janeiro State was tested using the pooling strategy. A total of 6096 samples were processed at the SESI Innovation Center for Occupational Health, constructing 1524 pools (Table 2). From those pools, 365 were positive (24.0%), which resulted in more 1460 RT-qPCR tests to identify the positive samples. Overall, an economy of 51.1% was observed using this strategy (Table 2). This result agrees with the statistical modeling prediction performed (Table 1).

**Table 2:** The pooling strategy applied to the testing program for industrial workers of Rio de Janeiro State.

PCR pool testing (1:4)	
People tested	6096
N pools tested	1524
N positive pools (%)	365 (24.0)
Total qRT-PCR runs^a^	2984
Population prevalence (%)	430 (7.0)
Cost savings (%)	US$66,171.96 (51.1)

^a^The data represents the total individual and pooling tests performed between April and May 2020.

### Development of a multiplex qPCR-based approach for the SARS-CoV-2 diagnosis in pooling samples

**Figure 3:**
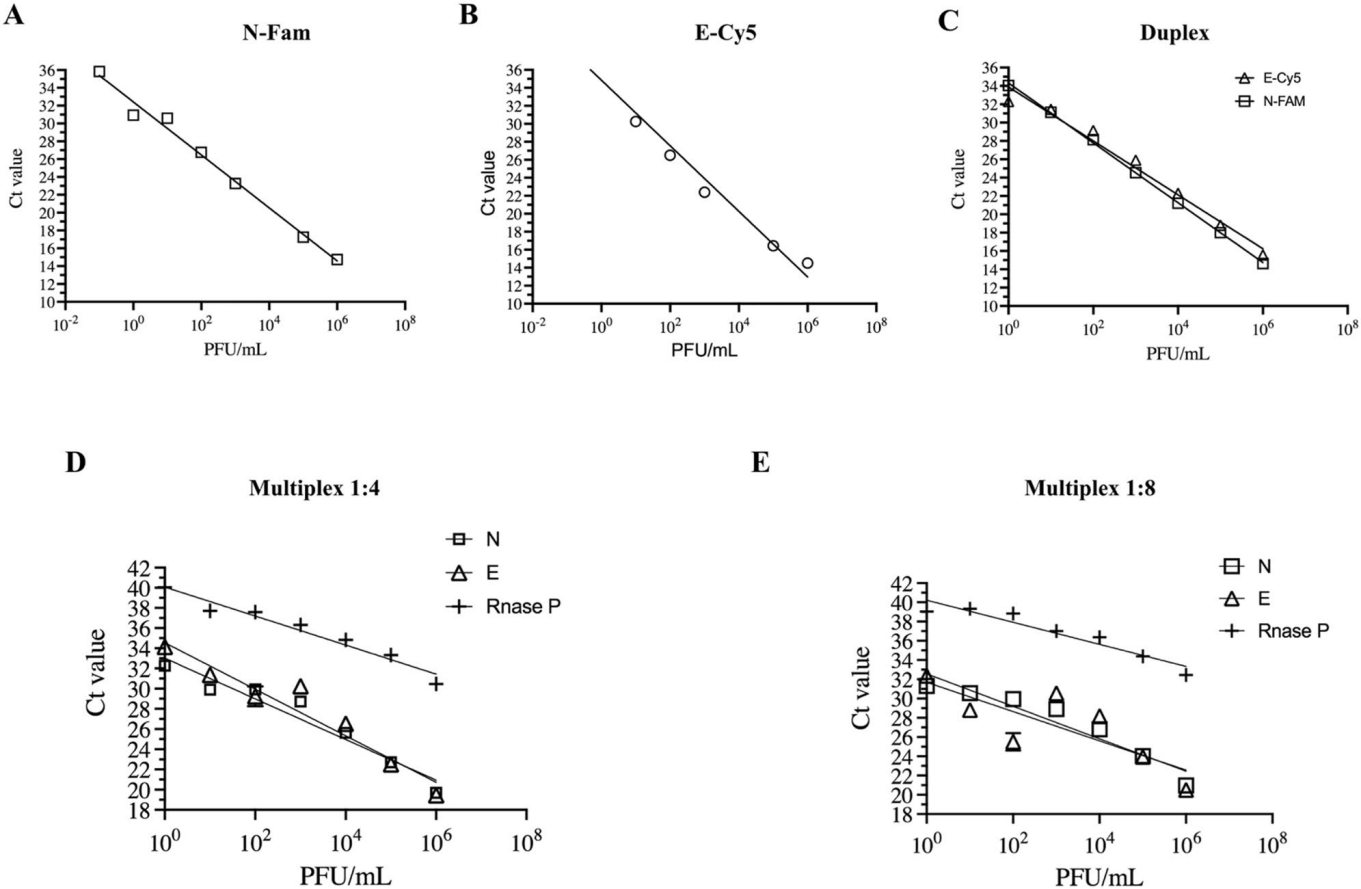
Validation of the multiplex/pooling strategy. Primers targeting genes N and E were evaluated separately (**A** and **B**), and in combination (**C**) using only an isolated virus stock. Multiplex combinations were evaluated combining an isolated virus stock with 4 (**D**) and 8 (**E**) clinical samples identified as negative to SARS-CoV-2.

Since our results with pooling samples maintained the sensitivity when using the singleplex CDC assay, we decided to evaluate if this strategy could maintain sensitivity while using a multiplex assay. When we analyze the efficiency of the multiplex RT-qPCR, by itself, the efficiency of each target detected alone (Fig. 3A,B and Table 3) was equivalent to the efficiency of detecting two viral targets in combination (Fig. 3C and Table 3) and two viral targets and the RNase P together (Fig. 3D,E and Table 3).

**Table 3:** Sensitivity of probes and its combinations for multiplex assay.

Targets (probe dye)	Sensitivity (%)
N (FAM)	99.78
E (FAM)	99.1
E (Cy5)	99.94
Orf1ab (FAM)	99.78
N (FAM)/E (Cy5)	99.78/99.94
N (FAM)/Orf1ab (Cy5)	99.97/99.98
E (FAM)/Orf1ab (Cy5)	99.58/99.97

The limit of detection (LOD) however, varied amongst the different primers and probe combinations, with E-Cy5 alone or in combination with N-FAM presenting the lowest LOD (0.1 PFU/mL) and the Orf1ab-Cy5 showing the highest LOD (100 PFU/mL alone and 10 PFU/mL in combination with both N-FAM and E-FAM) (Fig. 3B and Fig. S4A,B). When different dilutions of RNA extracted from infectious viruses were used in mixtures of 1:4 and 1:8 with RT-qPCR negative swab samples, the best efficiency and the lowest LOD was observed for the N-FAM/E-Cy5/RNaseP-Hex combination both in 1:4 to 1:8 proportions of viral RNA to negative swab samples (Fig. 3C,D and Fig. S4C–F). The RNase P gene amplification was interfered by a possible interaction with primers and/or probes from the combination E-Cy5/ Orf1ab-FAM/ RNaseP-Hex (Fig. S4C–F).

The N-FAM/ E-Cy5/ RNaseP-Hex combination gave the best results for RNaseP detection when compared to the N-FAM/ Orf1ab-Cy5/ RNaseP-Hex in the 1:8 proportion (Fig. 3D and Fig. S4E; Table S3). Therefore, a multiplex using the N-FAM/ E-Cy5/ RNaseP-Hex combination performs well in a pooling of both 4 and 8 samples/pool (Fig. 3C,D).

### Validation of N-FAM/E-Cy5/RNaseP-Hex multiplex alone and combined with pooling strategy for clinical samples

**Figure 4:**
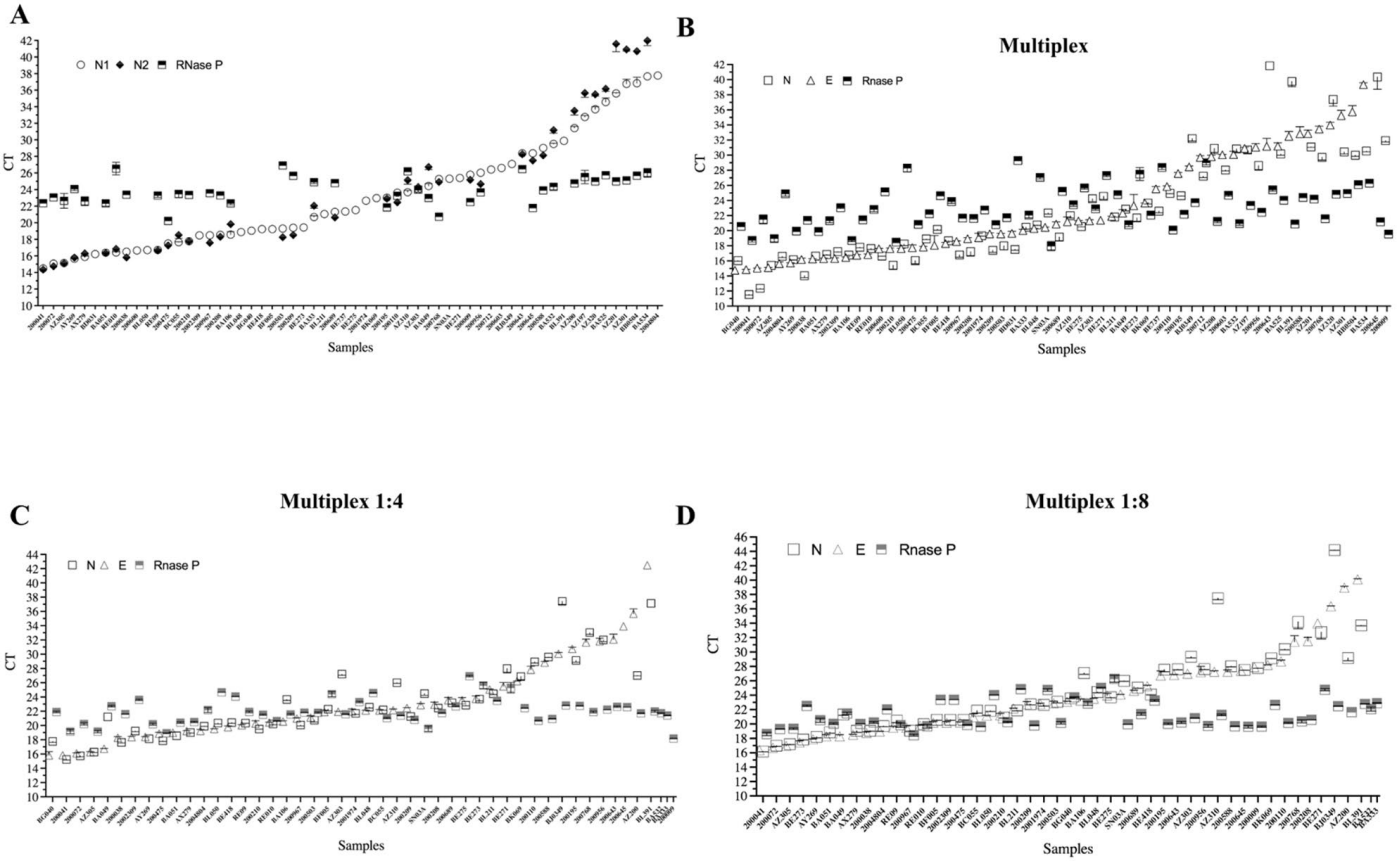
Multiplex N-Fam/E-Cy5/RNaseP panel validation using clinical samples. A total of 38 samples were evaluated from CDC singleplex using the N1 and N2 viral targets and the human RNaseP (**A**), the triplex (**B**), and combining multiplex and pooling strategies using 4 (**C**) and 8 (**D**) clinical samples.

The N-FAM/E-Cy5/RNaseP-Hex multiplex was the most efficient combination in the previous assays, therefore was chosen to be used in clinical samples. The sensitivity of the multiplex-pooling strategy was evaluated with 38 individual samples (Fig. 4; Table 3). Previously, we quantified these samples using the singleplex strategy recommended by the CDC^8^ (Fig. 4A). The triplex results showed that it is possible to detect SARS-CoV-2 even in samples with C_t_ values higher than 30 (Fig. 4B; Table S3). Considering the CDC criteria, for positive samples with C_t_s below 40 in both viral targets, the sensitivity of the triplex with the N-FAM/E-Cy5 /RNaseP-Hex combination was 84.2% (32/38) (Fig. 4B; Table S3). Pools of four and eight samples gave surprisingly the same sensitivity (89.47%) considering all C_t_ ranges (Fig. 4C,D; Table S3). Looking specifically at the C_t_ values up to 26, the multiplex pooling strategy had a sensitivity of 96.66% (Fig. 4C,D; Table S3). A total of 38 samples pooled gave positive signals for SARS-CoV-2 even when using an 8-specimen pool, without considerable sensitivity loss in the same C_t_ range (Fig. 4B,D; Table S3). Our results demonstrated that multiplex pooling of up to eight samples using N-FAM/E-Cy5/RNaseP-Hex gives the best results in samples with C_t_ values up to 31 (Fig. 4B,D; Table S3).

## Discussion

Diagnosis of SARS-Cov-2 remains a crucial factor even with the decrease of infections in several countries due to vaccination campaigns. At this point of the pandemic, the rapid detection of new cases and the surveillance needed for restoring normal activities are important for monitoring new waves of infection. Cheng and collaborators demonstrated that mass testing for contact tracing combined with phylogenetic analysis prevent new infections in Hong Kong besides its contributions to genomic surveillance of new SARS-CoV-2 variants^13^.

There are some options available for SARS-CoV-2 diagnostics that rely on the detection of viral proteins (antigen rapid tests) or viral genome (RT-qPCR). However, RT-qPCR is still the gold standard tool due to its high sensitivity and specificity. But for testing large populations, it becomes a limited technique because of the vast number of tests required and the deficit of reagents, equipment, and consumables in diagnostic laboratories around the world^14,15^.

Dorfman designed the strategy of pooling samples in 1940 to screen for syphilis infection in large populations of soldiers^16^. Since then, pooling is widely used for the detection of other pathogens for diagnostic purposes and even to study the prevalence of these agents in a defined population^10,11,17,18^. In a pandemic situation, as for COVID-19, pooling samples may be an interesting approach to overcome limitations related to large-scale diagnosis and provide access to mass testing in order to provide multi-time point surveillance and to define the prevalence rates in a certain region^19,20^.

One of the main concerns of pooling is the size of assembled pools that should be evaluated according to the prevalence of the pathogen in the study population. Some studies have demonstrated that for diseases with low population prevalence, this approach has the most potential for enabling mass testing at low costs, including for SARS-CoV-2 detection^12,21^. Besides, it was seen a great potential of pooling for repeat testing of the same population on a consecutive period, which could be an efficient strategy for disease control^18^. However, the prediction of ideal pool size (Table 1) requires a discerning in silico analysis otherwise the cost-saving will not be reached.

Mathematically, applying the Dorfman's approaches may incur savings as high as 90% within populations with a prevalence close to 1%. Our study adopted the statistical modeling approach and validated the data with pooling biological samples for COVID-19 diagnostic, confirming that the pool size must be selected according to the prevalence rate of positive cases in the population (Fig. 2). Besides, this combined analysis is essential to allow the optimization of limited resources and to apply mass testing enabling the management and reduction of underreporting, observed globally, but especially in large and developing countries^22,23^. For SARS-CoV-2 detection, other published studies performed pooling test validations based on mathematical models. Abdalhamid et al. used a web-based application to calculate pool size and reach similar results to ours with a recommended pool size of five samples for a 5% prevalence rate and an economy of 57% in tests^12^.

Although Yelin et al. have demonstrated the possibility of making pools using RNA or swab samples with the same test quality, the authors used a limited number of samples^24^. Here, our results showed that pooling RNA or nasopharyngeal swab samples have the same efficiency for SARS-CoV-2 diagnostic, which chooses pooling nasopharyngeal swab samples to overcome the RNA extraction bottleneck. Another critical factor is sample dilution that might be responsible for inconclusive or even false-negative results, depending on the C_t_ range of the analyzed samples. In this work, we thoroughly tested different concentrations of viral stock preparations and clinical samples in pools up to 32 samples. We demonstrated that even at the concentrations close to the RT-qPCR detection limit (pools up to 8 samples) were still positive. Larger pools could also lead to problems related to samples with low viral load, whose detection could be missed, and the logistics of assembly and deconvolution of these pools needs to be done carefully to avoid cross-contamination. A study proposes the deconvolution of pools divided into stages according to prevalence rate could optimize the process and increase the samples pooled^25^. Nevertheless, adding more steps to the diagnostic chain could delay the final diagnostic and spend more resources.

Our study associates in silico analyses and test validation to ensure a safe methodology to be widely used, and one that will reduce false and inconclusive diagnostic results and save costs. We performed the implementation of pool methodology in the COVID-19 mass testing program of the Industry Federation of the State of Rio de Janeiro. We observed substantial gains by reducing the qRT-PCR run time and the use of reagents and general consumables (Table 2). The statistical model predicted a cost saving of 48% (Table 1) for the pooling of four nasopharyngeal samples in a population of 7.5% of the prevalence rate. Indeed, we are observing an economy of 51.1% in our test routine. This cost saving already considers all the subsequent re-testing for the diagnosis of individual samples and the false-negative rate. In our analysis, the false-positive rate was 15.28%, meaning that 57 of 373 pools that presented a CT curve were negative when samples were analyzed individually. This rate is higher than previously reported by other authors^26,27^ but could be explained by factors that could impact on sample’s quality and stability, since it has already been demonstrated that oro/nasopharyngeal sample’s freeze–thaw and time of storage since sample collection reduce viral load^28–30^. In our case, samples were stored at − 80 °C within 4 h of initial handling and thaw for individual testing. Also, factors associated with mixing of several nasopharyngeal samples within a reaction tube which is diluted out when testing the sample individually, could be considered. For instance, we established a stringent parameter for the pooling test interpretation, for which any positive signal regardless of the C_t_ and the fluorescence signal was considered positive, even those that would be considered inconclusive in individual tests. Therefore, excluding results with C_t_ values above 34 in the pooling test, the rate drops to 5.19%, it is within the range of other studies^26,27^. Given that the test cost of COVID-19 diagnostic in-house PCR used in our company is around US$22.20 per individual sample, pooling samples by four reduces the experimental cost to US$10.85 per test. This reduction of approximately twice was also observed in another study for a prevalence of 5%^26^.

The pooling strategy is already fairly established for COVID-19 mass testing in some countries such as Germany and India. Our study shows that combining this strategy with *in-silico* analyses will improve its use. To increase the added value, the combination of sample pooling strategy and a multiplex RT-qPCR was predicted to promote an economy of 47.29 and 25.63% for pools of 4 and 8 samples respectively, according to the model predicted for a COVID-19 prevalence rate of 7.8%.

Ishige et al. described that the multiplex N + E is a good combination when evaluated with the internal control hABL1^31^. Another study corroborated this result using 27 samples in blind testing^32^. They compared singleplex reactions with multiplexes using hRNase P as an internal control. Among them, 12 samples (44.4% of positive rate) were positives in singleplex configuration and 11 in the multiplex, with no sensitivity loss^32^. Another report developed a multiplex assay using the CDC N1 and N2 primers modified with different probes and demonstrated a limit of detection of 50 copies/reaction^33^. This study also analyzed clinical samples and the multiplex sensitivity was conserved when compared to CDC singleplex. However, as previously reported^34^, the N2 primer showed a reduced sensitivity. In this way, a multiplex assay that uses two distinct viral targets would be useful. Despite these promissory findings, we decide to evaluate three possible multiplex combinations, associating with sample pooling strategy. We demonstrated that the pooling approach using 4 or 8 samples tested with a multiplex combination in RT-qPCR is feasible to be applied without sensitivity loss, mainly combining Nucleocapsid (N) and Envelope (E) genes (Fig. 4B) while for the other combinations of target genes evaluated, the sensitivity was lower (Table S3).

A recent study that combined pooling samples and multiplex used only a pool of four samples and the multiplex was performed using the CDC primers only for viral N gene and human RNaseP. Despite this difference compared to our study, it was also observed a high concordance between singleplex and multiplex results with samples with higher C_t_ values presenting discordant results^35^.

In summary, our study demonstrates that the implementation of pooling strategy combined with a multiplex assay can boost testing efficiency, save resources, and reduce costs while maintaining test sensitivity. A robust in-silico analysis is useful to analyze the prevalence of positive cases in a certain region and supports the design of the best pooling strategy to be used in this population. In populations with low prevalence of positive cases, this approach could help to implement mass testing and detect the transmission of SARS-CoV-2 in the community, supporting actions to control the spread of the virus.

## Methods

### Samples

A total of 152 clinical samples were selected from the mass testing program of Industry Federation of the State of Rio de Janeiro (Brazil), from industry workers, between April and May 2020. The nasopharyngeal swabs were conditioned in 2.0 mL of DMEM medium (Thermo Fisher), and 1.5 mL of each sample were individually stored at − 80 ºC in cryotubes until further use. For the pooling validation, we used leftovers from routine testing samples, and no personal, clinical, and demographic data from individuals were accessed or released. The National Committee of Research Ethics (CAAE 36602620.6.0000.5257) reviewed and approved the present study. All methods and experimental protocols were approved and carried out in accordance with guidelines and regulations from the institutional committee.

#### Sensitivity tests

SARS-CoV-2 A2 isolate (GISAID: 528539) used to detect the maximum sensitivity of the assays during pooling and multiplex analyses. The total amount of 10^6^ viral infectious particles per mL was serially diluted (10^–1^, 10^–2^, 10^–3^, 10^–4^, 10^–5^, 10^–6^, 10^–8^, 10^–9^ and 10^–10^ infectious viral particles/mL) were pooled with nasopharyngeal swabs previously diagnosed as negatives for COVID-19.

To determine the sensitivity of different pool sizes (4, 8, and 16 samples each), different amounts of the SARS-CoV-2 A2 isolate (100, 1000, and 10,000 viral infectious particles/mL) were pooled with nasopharyngeal swabs by SARS-CoV-2 qRT-PCR using targets different from the initial test. For the multiplex strategy to address combined multiplex and pooling sensitivity, we used several dilutions of viral RNA extracted from isolated viruses from tissue culture, and combined with pooling strategies.

### Pooling assembly strategies

Here, it was performed a comparative analysis between the nasopharyngeal sample and RNA pooling approaches to identify the best method to construct the pools.

#### Nasopharyngeal sample pooling

To evaluate the effect of pooling samples on the qRT-PCR sensitivity, Cycle Threshold (CT) values of pools were compared with CT values of the individual test at the same run. A total of 38 patients previously described as positive with distinct CT values were tested in the pooling test using frozen aliquots of nasopharyngeal swabs previously detected as positive or negative in the diagnostic tests. Each pool was prepared with an equal volume of one positive and others *n* negative patients, varying according to the pool size. Combinations of 4, 8, 16, or 32 specimens were tested. The final volume of 300 µL was used to perform RNA extraction.

#### RNA pooling

For RNA pools, we selected 24 RNA samples individually extracted from nasopharyngeal swabs before the pool's construction. Pooling was performed from RNA samples, combining one SARS-CoV-2 positive with another *n* negative RNAs, ranging according to the pool size. As well as sample pooling, we tested pools of 4, 8, 16, and 32 RNA sample combinations.

### Multiplex assembly strategy

Clinical nasopharyngeal swabs samples previously tested for SARS-CoV-2 using the standard CDC protocol^8^, were used to evaluate the performance of the proposed multiplex and pooling combined strategy. These samples were chosen based on the cycle threshold (CT) values ranging from 15 to 25. A total of 38 samples for N-FAM/ E-Cy5/hRNaseP-Hex combination, and 14 for triplexed N-FAM/Orf1ab-Cy5/hRNaseP-Hex and E-FAM/ Orf1ab-Cy5/hRNaseP-Hex were pooled using one specimen previously detected as positive and three or seven negative specimens (1:4 and 1:8, respectively).

### RNA extraction

RNA extraction was performed mechanically by Maxwell^®^ RSC (Promega) using a sample volume of 300 μL. Total RNA was obtained by the Maxwell^®^ RSC Viral Total Nucleic Acid Purification Kit (catalog number: AS1330-Promega) according to the manufactured protocol. Non-pooled samples and negative control also had RNA extracted.

### cDNA synthesis and real-time PCR

qRT-PCR reactions were performed in one step with Brilliant III Ultra-Fast qRT-PCR Master Mix (catalog number: 600884—Agilent). Each reaction containing: 10 μL of 2X QRT-PCR Master Mix; 1.5 μL prime time; 0.2 μL of 100 mM DTT; 0.3 of reference dye (dilute 1:500); 1.0 μL of RT/RNase block; 2.0 μL of nuclease-free water and 5 μL of RNA. Reactions were cycled at QuantStudio 5 (Thermo Scientific) according to the following program: 50 °C for 10 min; 95 °C for 3 min; 45 cycles of 95 °C for 3 s and 53 °C for 30 s.

### Primers and probes

For pooling strategy analysis primers and probes for viral N gene (N1 and N2) were synthesized as primetime chemistry by Integrated DNA Technologies (IDT) according to sequences from the Center for Disease Control and Prevention (CDC-US). Human RNase P (hRNase P) was used as human specimen control.

For multiplex strategy, primers and probes for Envelope (E—primers: Gene E_sarbeco_F1 5′ ACA GGT ACG TTA ATA GTT AAT AGC GT 3′; Gene E_sarbeco_R2 5′ ATA TTG CAG CAG TAC GCA CAC A 3′; and probe: E_sarbeco_P1 5′ ACA CTA GCC ATC CTT ACT GCG CTT CG 3′ with dye FAM or Cy5), Nucleocapsid (N—primers: 2019-nCoV_N1_F 5′ GAC CCC AAA ATC AGC GAA AT 3′; 2019-nCoV_N1_R 5′ TCT GGT TAC TGC CAG TTG AAT CTG 3′; and probe 2019-nCoV_N1_P 5′ ACC CCG CAT TAC GTT TGG TGG ACC 3′ with dye FAM) and Orf1ab (primers: Orf1ab 1ab_F 5′ CTC TGG AAC ACT TTT ACA AGA CTT C 3′; Orf1ab 1ab_R 5′ ACC ATC AAC TTT TGT GTA AAC AGT G 3′; and probe Orf1ab 1b_P2 5′ ACA GGG TGA AGT ACC A with dye Cy5) were synthetized separately. The combinations tested in order to identify the most reliable multiplex combination were: N + E, N + Orf1ab and E + Orf1ab along with the human gene internal control hRNase P (primers: hRNaseP6_R 5′ GAG CGG CTG TCT CCA CAA GT 3′; hRNaseP6_F 5′ AGA TTT GGA CCT GCG AGC 3′; and probe hRNaseP6_P 5′ TTC TGA CCT GAA GGC TCT GCG CG 3′ with dye Hex). All probes presented Iowa Black as quencher. The qPCR assays were performed in triplicates.

### Statistical analysis

Principal Component Analysis (PCA) was performed by using Unscrambler Software (version 10.1; CAMO AS; Trondheim, Norway). Grouping pattern was investigated within samples S23-S38, considering as variables the pool type (sample pooling or RNA pooling) and the pool size (4, 8, 16, or 32 samples).

### In-silico experimental design using pooling methodology

Mathematical modeling of the pooling strategy was performed based on pool size and prevalence of positive cases in population. Each pooled test was modeled as a Bernoulli trial and with the probability of a positive test *p* based on the results obtained from the clinical nasopharyngeal swab samples collected from industrial workers. The standard case was no positive cases within a pooled test, given by:

1 $$P\left(neg\right)= {(1-p)}^{n}$$

where P(*neg*) gives the probability (*p*) of a given pooled sample with size (*n*) having no positives based on the exponential distribution.

To estimate the cost savings related to a pooling strategy, the model below (2)

2 $$V\left(v,p,n\right)= \frac{v}{n}+v\left(1-P\left(neg\right)\right)$$

was developed to estimate the reduced test value using the pooling strategy, where *v* represents the costs for a single test, *p* is the probability of a positive test, *n* is the selected pool size, and the term *v*(1 − P(*neg*)) gives the cost of redoing tests given P(*neg*).

The cost savings are given by the ratio between the reduced test value and the single test value subtracted from the whole.

### Pooling methodology application in clinical samples

To validate the in silico experimental design developed in this work, as well as increase the testing capacity performed by SESI Innovation Center for Occupational Health, clinical samples collected individually, as described above, were pooled using four specimens. A total of 6,096 samples were tested for SARS-CoV-2 using the RT-PCR processed as 1524 pools (1:4).

### Pooling test and individual test interpretation samples

In our routine, we established the CDC criteria for individual tests^7^. However, for the pooling strategy, detection of any fluorescence signal in a pool, regardless of the CT or fluorescence value, was considered positive, and the samples in the specific pool were analyzed individually.

### Ethical declaration

The present study was approved by the National Committee of Research Ethics (CAAE 36602620.6.0000.5257). All enrolled participants were over 18 years old and declared written informed consent.

## Supplementary Information

Supplementary Information.

## Data Availability

The datasets generated and/or analyzed during the current study are available in the BioModels repository, under the identifier MODEL2201260001. It can be also accessed through the following weblink: (https://www.ebi.ac.uk/biomodels/MODEL2201260001).

## References

[1] Zhou P, Yang XL, Wang XG, Hu B, Zhang L, Zhang W, Si HR, Zhu Y, Li B, Huang CL, Chen HD, Chen J, Luo Y, Guo H, Jiang RD, Liu MQ, Chen Y, Shen XR, Wang X, Zheng XS, Zhao K, Chen QJ, Deng F, Liu LL, Yan B, Zhan FX, Wang YY, Xiao GF, Shi ZL (2020). A pneumonia outbreak associated with a new coronavirus of probable bat origin. Nature.

[2] World Health Organization. Coronavirus Disease (COVID-19) Dashboard. (2021). https://covid19.who.int/. Accessed 30 November 2021.

[3] H. Ritchie, E. Mathieu, L. Rodés-Guirao, C. Appel, C. Giattino, E. Ortiz-Ospina, J. Hasell, B. Macdonald, D. Beltekian & M. Roser (2020) "Coronavirus Pandemic (COVID-19)". Published online at OurWorldInData.org. https://ourworldindata.org/coronavirus. [Online Resource]. Accessed 30 November 2021.

[4] World Health Organization. Considerations in adjusting public health and social measures in the context of COVID-19. (2021). WHO/2019-nCoV/Adjusting_PH_measures/2021.1.

[5] Salathé M, Althaus CL, Neher R, Stringhini S, Hodcroft E, Fellay J, Zwahlen M, Senti G, Battegay M, Wilder-Smith A, Eckerle I, Egger M, Low N (2020). COVID-19 epidemic in Switzerland: On the importance of testing, contact tracing and isolation. Swiss Med Wkly..

[6] Rate of coronavirus (COVID-19) tests performed in the most impacted countries worldwide as of July 22, 2020 (per million population). Health & Pharmaceuticals (2020). https://www.statista.com/statistics/1104645/covid19-testing-rate-select-countries-worldwide/. Accessed 24 October 2021.

[7] World Health Organization. (2020). Clinical management of COVID-19: Interim guidance, 27 May 2020. World Health Organization. https://www.who.int/publications/i/item/considerations-in-adjusting-public-health-and-social-measures-in-the-context-of-covid-19-interim-guidance. Accessed 14 June 2021.

[8] CDC 2019-Novel Coronavirus (2019-nCoV) real-time RT-PCR diagnostic panel. (2020). 10.1371/journal.pone.0260487 PMC8673615 34910739

[9] Corman VM, Landt O, Kaiser M, Molenkamp R, Meijer A, Chu DK, Bleicker T, Brünink S, Schneider J, Schmidt ML, Mulders DG, Haagmans BL, van der Veer B, van den Brink S, Wijsman L, Goderski G, Romette JL, Ellis J, Zambon M, Peiris M, Goossens H, Reusken C, Koopmans MP, Drosten C (2020). Detection of 2019 novel coronavirus (2019-nCoV) by real-time RT-PCR. Euro Surveill..

[10] Emmanuel JC, Bassett MT, Smith HJ, Jacobs JA (1988). Pooling of sera for human immunodeficiency virus (HIV) testing: An economical method for use in developing countries. J. Clin. Pathol..

[11] Van TT, Miller J, Warshauer DM, Reisdorf E, Jernigan D, Humes R, Shult PA (2012). Pooling nasopharyngeal/throat swab specimens to increase testing capacity for influenza viruses by PCR. J. Clin. Microbiol..

[12] Abdalhamid B, Bilder CR, McCutchen EL, Hinrichs SH, Koepsell SA, Iwen PC (2020). Assessment of specimen pooling to conserve SARS CoV-2 testing resources. Am. J. Clin. Pathol..

[13] Cheng VC, Siu GK, Wong SC, Au AK, Ng CS, Chen H, Li X, Lee LK, Leung JS, Lu KK, Lo HW, Wong EY, Luk S, Lam BH, To WK, Lee RA, Lung DC, Kwan MY, Tse H, Chuang SK, To KK, Yuen KY (2021). Complementation of contact tracing by mass testing for successful containment of beta COVID-19 variant (SARS-CoV-2 VOC B.1.351) epidemic in Hong Kong. Lancet Reg. Health West Pac..

[14] Du Z, Pandey A, Bai Y, Fitzpatrick MC, Chinazzi M, Pastore Y, Piontti A, Lachmann M, Vespignani A, Cowling BJ, Galvani AP, Meyers LA (2021). Comparative cost-effectiveness of SARS-CoV-2 testing strategies in the USA: A modelling study. Lancet Public Health..

[15] Neilan, A.M., Losina, E., Bangs, A.C., Flanagan, C., Panella, C., Eskibozkurt, G.E., Mohareb, A., Hyle, E.P., Scott, J.A., Weinstein, M.C., Siedner, M.J., Reddy, K.P., Harling, G., Freedberg, K.A., Shebl, F.M., Kazemian, P., Ciaranello, A.L. Clinical impact, costs, and cost-effectiveness of expanded severe acute respiratory syndrome coronavirus 2 testing in Massachusetts. *Clin. Infect. Dis*. **73**(9), e2908–e2917 (2021). 10.1093/cid/ciaa1418. 10.1093/cid/ciaa1418 PMC7543346 32945845

[16] Dorfman R (1943). The detection of defective members of large populations. Ann. Math. Stat..

[17] Shipitsyna E, Shalepo K, Savicheva A, Unemo M, Domeika M (2007). Pooling samples: The key to sensitive, specific and cost-effective genetic diagnosis of Chlamydia trachomatis in low-resource countries. Acta Derm. Venereol..

[18] Lindan C, Mathur M, Kumta S, Jerajani H, Gogate A, Schachter J, Moncada J (2005). Utility of pooled urine specimens for detection of Chlamydia trachomatis and Neisseria gonorrhoeae in men attending public sexually transmitted infection clinics in Mumbai, India, by PCR. J. Clin. Microbiol..

[19] Du Z, Pandey A, Bai Y, Fitzpatrick MC, Chinazzi M, Pastore Y, Piontti A, Lachmann M, Vespignani A, Cowling BJ, Galvani AP, Meyers LA (2021). Comparative cost-effectiveness of SARS-CoV-2 testing strategies in the USA: A modelling study. Lancet Public Health.

[20] Pilcher CD, Westreich D, Hudgens MG (2020). Group testing for severe acute respiratory syndrome-coronavirus 2 to enable rapid scale-up of testing and real-time surveillance of incidence. J. Infect. Dis..

[21] Borillo GA, Kagan RM, Baumann RE, Fainstein BM, Umaru L, Li HR, Kaufman HW, Clarke NJ, Marlowe EM (2020). Pooling of upper respiratory specimens using a SARS-CoV-2 real-time RT-PCR assay authorized for emergency use in low-prevalence populations for high-throughput testing. Open Forum Infect. Dis..

[22] Saraniti BA (2006). Optimal pooled testing. Health Care Manage Sci..

[23] Sinnott-Armstrong, N., Klein, D.L., Hickey, B. Evaluation of Group Testing for SARS-CoV-2 RNA. medRxiv. 2020.03.27.20043968. 10.1101/2020.03.27.20043968.

[24] Yelin I, Aharony N, Tamar ES, Argoetti A, Messer E, Berenbaum D, Shafran E, Kuzli A, Gandali N, Shkedi O, Hashimshony T, Mandel-Gutfreund Y, Halberthal M, Geffen Y, Szwarcwort-Cohen M, Kishony R (2020). Evaluation of COVID-19 RT-qPCR test in multi sample pools. Clin. Infect. Dis..

[25] Eberhardt JN, Breuckmann NP, Eberhardt CS (2020). Multi-stage group testing improves efficiency of large-scale COVID-19 screening. J. Clin. Virol..

[26] Wacharapluesadee S, Kaewpom T, Ampoot W, Ghai S, Khamhang W, Worachotsueptrakun K, Wanthong P, Nopvichai C, Supharatpariyakorn T, Putcharoen O, Paitoonpong L, Suwanpimolkul G, Jantarabenjakul W, Hemachudha P, Krichphiphat A, Buathong R, Plipat T, Hemachudha T (2020). Evaluating the efficiency of specimen pooling for PCR-based detection of COVID-19. J. Med. Virol..

[27] Handous I, Hannachi N, Marzouk M, Hazgui O, Bouafif Ep Ben Alaya N, Boukadida J (2021). Pooling nasopharyngeal swab specimens to improve testing capacity for SARS-CoV-2 by real-time RT-PCR. Biol. Proced. Online..

[28] Liotti FM, Menchinelli G, Marchetti S, Morandotti GA, Sanguinetti M, Posteraro B, Cattani P (2021). Evaluation of three commercial assays for SARS-CoV-2 molecular detection in upper respiratory tract samples. Eur. J. Clin. Microbiol. Infect. Dis..

[29] Pujadas E, Ibeh N, Hernandez MM, Waluszko A, Sidorenko T, Flores V, Shiffrin B, Chiu N, Young-Francois A, Nowak MD, Paniz-Mondolfi AE, Sordillo EM, Cordon-Cardo C, Houldsworth J, Gitman MR (2020). Comparison of SARS-CoV-2 detection from nasopharyngeal swab samples by the Roche cobas 6800 SARS-CoV-2 test and a laboratory-developed real-time RT-PCR test. J. Med. Virol..

[30] Oguri S, Fujisawa S, Kamada K, Nakakubo S, Yamashita Y, Nakamura J, Horii H, Sato K, Nishida M, Teshima T, Ohiro Y, Takada A, Konno S (2021). Effect of varying storage conditions on diagnostic test outcomes of SARS-CoV-2. J. Infect..

[31] Ishige T, Murata S, Taniguchi T, Miyabe A, Kitamura K, Kawasaki K, Nishimura M, Igari H, Matsushita K (2020). Highly sensitive detection of SARS-CoV-2 RNA by multiplex rRT-PCR for molecular diagnosis of COVID-19 by clinical laboratories. Clin. Chim. Acta..

[32] Waggoner JJ, Stittleburg V, Pond R, Saklawi Y, Sahoo MK, Babiker A, Hussaini L, Kraft CS, Pinsky BA, Anderson EJ, Rouphael N (2020). Triplex real-time RT-PCR for severe acute respiratory syndrome coronavirus 2. Emerg. Infect. Dis..

[33] Kudo E, Israelow B, Vogels CBF, Lu P, Wyllie AL, Tokuyama M, Venkataraman A, Brackney DE, Ott IM, Petrone ME, Earnest R, Lapidus S, Muenker MC, Moore AJ, Casanovas-Massana A, Omer SB, Dela Cruz CS, Farhadian SF, Ko AI, Grubaugh ND, Iwasaki A, Yale IMPACT Research Team (2020). Detection of SARS-CoV-2 RNA by multiplex RT-qPCR. PLoS Biol..

[34] Vogels CBF, Brito AF, Wyllie AL, Fauver JR, Ott IM, Kalinich CC, Petrone ME, Casanovas-Massana A, Catherine Muenker M, Moore AJ, Klein J, Lu P, Lu-Culligan A, Jiang X, Kim DJ, Kudo E, Mao T, Moriyama M, Oh JE, Park A, Silva J, Song E, Takahashi T, Taura M, Tokuyama M, Venkataraman A, Weizman OE, Wong P, Yang Y, Cheemarla NR, White EB, Lapidus S, Earnest R, Geng B, Vijayakumar P, Odio C, Fournier J, Bermejo S, Farhadian S, Dela Cruz CS, Iwasaki A, Ko AI, Landry ML, Foxman EF, Grubaugh ND (2020). Analytical sensitivity and efficiency comparisons of SARS-CoV-2 RT-qPCR primer-probe sets. Nat. Microbiol..

[35] Lu X, Sakthivel SK, Wang L, Lynch B, Dollard SM (2021). Enhanced throughput of the severe acute respiratory syndrome coronavirus 2 (SARS-CoV-2) real-time RT-PCR panel by assay multiplexing and specimen pooling. J. Virol. Methods..

