# Supplementary material for: Boosting SARS-CoV-2 detection combining pooling and multiplex strategies
Source: Sci Rep. 2022 May 23;12:8684. doi: 10.1038/s41598-022-12747-8 (PMC9126939; doi:10.1038/s41598-022-12747-8)
Supplement: Supplementary file 1 — Supplementary Information. [file 41598_2022_12747_MOESM1_ESM.docx]

**Supplementary information**

**Figures**

**
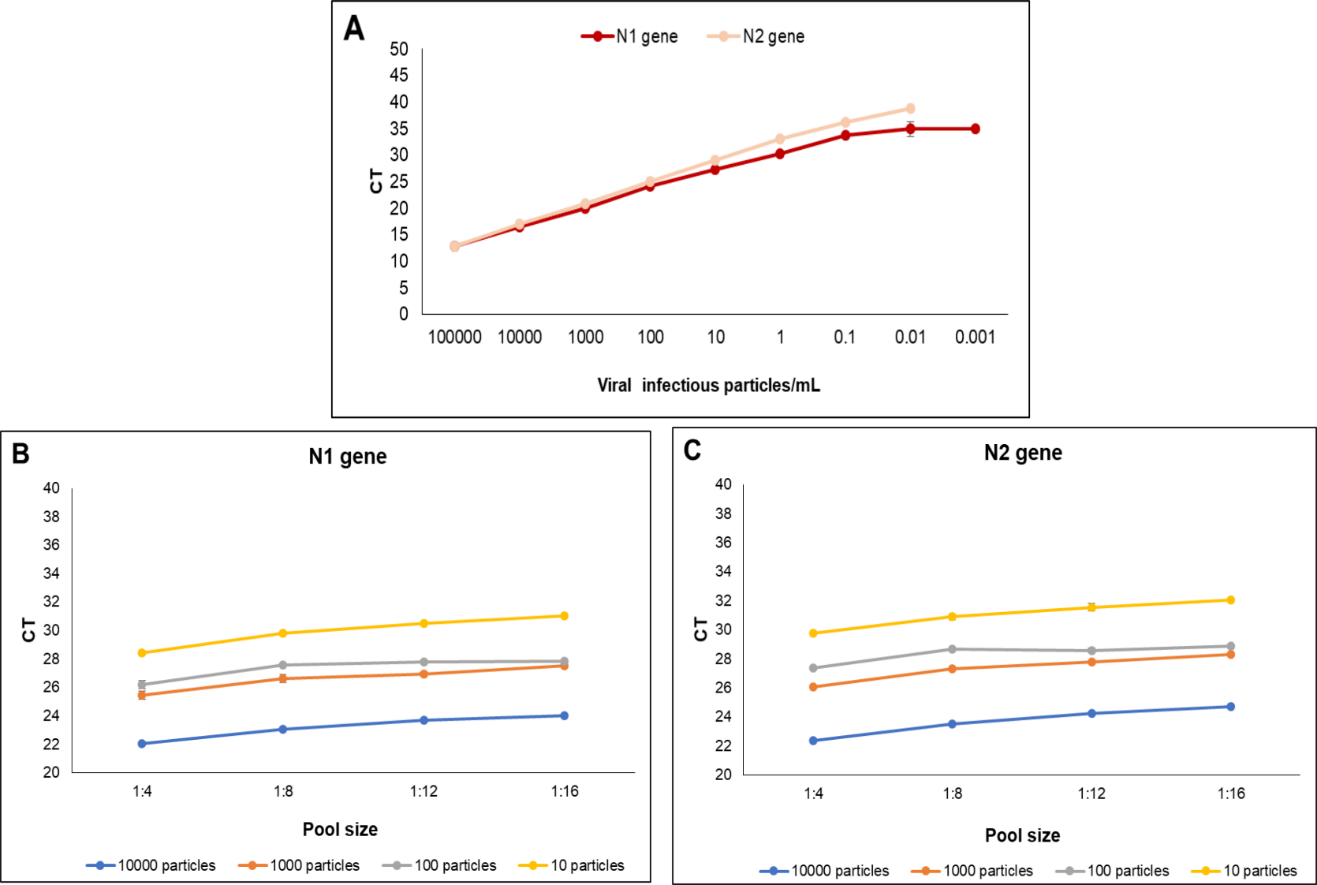
**

**Fig. S1.** Isolated virus testing. Limit of detection from SARS-CoV-2 using N1 and N2 genes (A); Curves of detection for pooling different titers of isolated virus (1:4 = isolated virus + 3 negative samples; 1:8 = isolated virus + 7 negative samples; 1:16 = isolated virus + 15 negative samples; 1:32 = isolated virus + 31 negative samples) for N1 (B) and N2 (C).


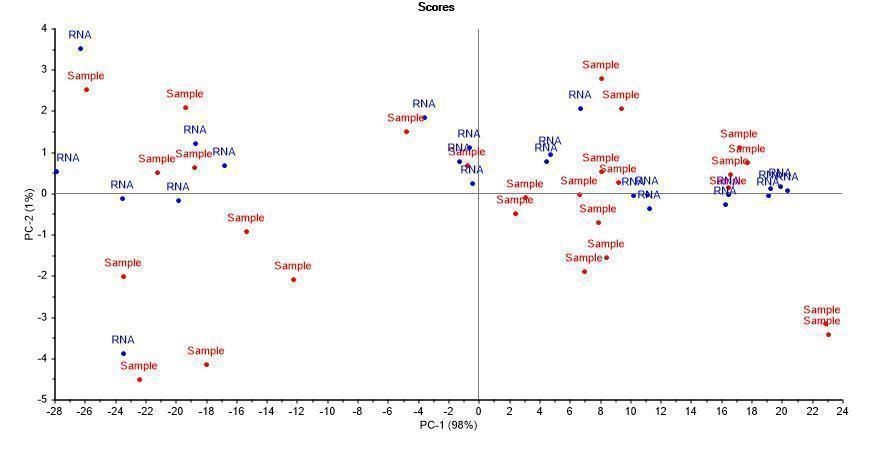


**Fig. S2.** Principal component analysis (PCA) between samples and RNA pooled.


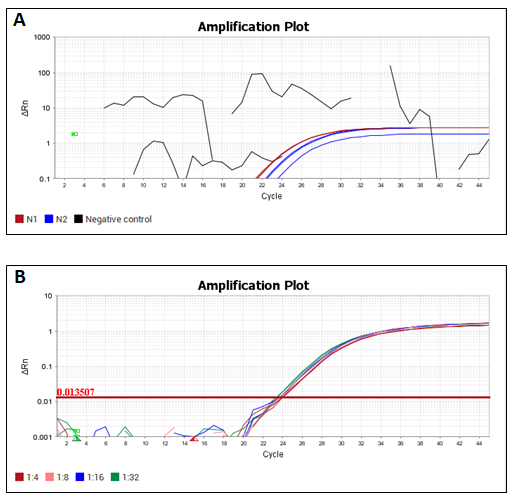


**Fig. S3.** Pooling tests used patient samples. (A) Positive and negative controls from N1 and N2; (B) Curves represent hRNase P gene (N1 and N2 genes not presented amplification). 1:4, 1:8, 1:16 and 1:32 correspond to one positive sample pooled with other 3, 7, 15 and 31 negative samples, respectively.

**Fig. S4.** Isolated virus testing in the multiplex assays. Limit of detection from SARS-CoV-2 for the N/Orfab (A) and E/Orfab (B) combinations. Curves of detection for pooling different titers of isolated virus using 4 (C and D) and 8 (E and F) clinical samples tested as negative.

**Tables**

**Table S1.** Pool of samples. Each mean sample represents one patient tested. Standard deviation (SD) represents the technical replicates Traces represent spots no evaluated. ND= no detectable.

| **Sample** | **N1** | | | | | | | | | **N2** | | | | | | | | |
| --- | --- | --- | --- | --- | --- | --- | --- | --- | --- | --- | --- | --- | --- | --- | --- | --- | --- | --- |
|  | **Absolute CT** | **Pool 1:4** | | **Pool 1:8** | | **Pool 1:16** | | **Pool 1:32** | | **Absolute CT** | **Pool 1:4** | | **Pool 1:8** | | **Pool 1:16** | | **Pool 1:32** | |
|  |  | **Mean** | **SD** | **Mean** | **SD** | **Mean** | **SD** | **Mean** | **SD** |  | **Mean** | **SD** | **Mean** | **SD** | **Mean** | **SD** | **Mean** | **SD** |
| S1 | 18.81 | 27.06 | 0.03 | 29.13 | 0.15 | - | - | - | - | - | 26.02 | 0.08 | 27.91 | 0.06 | - | - | - | - |
| S2 | 17.03 | 24.18 | 0.16 | 25.74 | 0.16 | - | - | - | - | - | 23.05 | 0.13 | 25.26 | 0.17 | - | - | - | - |
| S3 | 14.24 | 22.44 | 0.09 | 25.43 | 0.10 | - | - | - | - | - | 20.39 | 0.31 | 23.71 | 0.08 | - | - | - | - |
| S4 | 19.96 | 26.42 | 0.11 | 27.97 | 0.00 | - | - | - | - | - | 25.21 | 0.30 | 27.65 | 0.02 | - | - | - | - |
| S5 | 22.56 | 29.40 | 0.16 | 30.27 | 0.09 | - | - | - | - | - | 28.50 | 0.07 | 29.14 | 0.05 | - | - | - | - |
| S6 | 20.98 | 26.23 | 0.04 | 27.74 | 0.09 | - | - | - | - | - | 26.06 | 0.00 | 27.70 | 0.06 | - | - | - | - |
| S7 | 17.46 | 23.84 | 0.07 | 26.17 | 0.11 | - | - | - | - | - | 22.71 | 0.05 | 25.10 | 0.04 | - | - | - | - |
| S8 | 30.28 | 29.78 | 0.03 | 32.17 | 0.39 | - | - | - | - | - | 30.78 | 0.26 | 32.89 | 0.28 | - | - | - | - |
| S9 | 26.56 | 28.64 | 0.06 | 30.52 | 0.15 | - | - | - | - | - | 29.15 | 0.44 | 31.18 | 0.14 | - | - | - | - |
| S10 | 27.33 | 32.05 | 0.32 | 33.53 | 0.42 | - | - | - | - | - | 34.14 | 0.84 | 33.22 | 0.26 | - | - | - | - |
| S11 | 24.07 | 28.12 | 0.07 | 28.56 | 0.02 | - | - | - | - | - | 28.17 | 0.16 | 28.80 | 0.09 | - | - | - | - |
| S12 | 28.09 | 32.62 | 0.21 | 34.51 | 0.11 | - | - | - | - | - | 31.81 | 0.17 | 33.78 | 0.32 | - | - | - | - |
| S13 | 29.44 | 32.69 | 0.23 | 34.37 | 0.22 | - | - | - | - | - | 31.64 | 0.14 | 34.79 | 0.19 | - | - | - | - |
| S14 | 32.96 | 32.10 | 0.12 | 36.91 | 0.65 | - | - | - | - | - | 32.12 | 0.29 | 38.47 | 0.00 | - | - | - | - |
| S15 | 20.42 | 27.33 | 0.11 | 29.74 | 0.04 | - | - | - | - | - | 27.89 | 0.33 | 30.62 | 0.26 | - | - | - | - |
| S16 | 33.71 | 36.99 | 0.57 | 36.62 | 0.00 | - | - | - | - | - | 41.26 | 0.88 |  |  | - | - | - | - |
| S17 | 33.45 | 34.28 | 0.18 | 33.72 | 0.40 | - | - | - | - | - | 36.06 | 0.48 | 34.93 | 0.18 | - | - | - | - |
| S18 | 35.63 | 35.90 | 0.59 | 36.29 | 0.50 | - | - | - | - | - | 39.73 | 0.00 | 41.22 | 1.22 | - | - | - | - |
| S19 | 31.39 | 38.09 | 0.77 | 38.41 | 0.37 | - | - | - | - | - | 39.13 | 0.33 | 38.57 | 0.78 | - | - | - | - |
| S20 | 34.98 | 38.42 | 0.16 | 39.76 | 0.80 | - | - | - | - | - | 39.97 | 0.42 | 40.64 | 1.66 | - | - | - | - |
| S21 | 35.01 | 37.14 | 0.82 | 37.57 | 0.53 | - | - | - | - | - | 40.60 | 0.00 | 41.22 | 0.00 | - | - | - | - |
| S22 | 35.7 | 26.08 | 0.08 | 27.87 | 0.13 | - | - | - | - | - | 25.99 | 0.04 | 27.56 | 0.06 | - | - | - | - |
| S23 | 22.93 | 25.46 | 0.09 | 26.00 | 0.08 | 27.26 | 0.08 | 28.44 | 0.06 | 24.07 | 26.47 | 0.04 | 28.92 | 0.87 | 29.87 | 0.55 | 29.77 | 0.06 |
| S24 | 15.29 | 17.50 | 0.16 | 18.46 | 0.05 | 19.75 | 0.20 | 20.54 | 0.09 | 15.00 | 17.41 | 0.04 | 18.52 | 0.12 | 19.73 | 0.09 | 20.64 | 0.03 |
| S25 | 15.50 | 15.31 | 0.05 | 17.74 | 0.03 | 19.75 | 0.13 | 20.54 | 0.03 | 15.57 | 15.49 | 0.07 | 17.85 | 0.03 | 20.22 | 0.14 | 20.89 | 0.06 |
| S26 | 18.59 | 20.68 | 0.17 | 21.95 | 0.04 | 22.63 | 0.07 | 23.94 | 0.12 | 19.00 | 21.11 | 0.08 | 22.48 | 0.09 | 23.19 | 0.05 | 24.54 | 0.04 |
| S27 | 17.74 | 20.12 | 0.08 | 21.07 | 0.04 | 23.87 | 0.01 | 24.80 | 0.03 | 18.15 | 20.40 | 0.03 | 21.42 | 0.04 | 24.73 | 0.08 | 25.86 | 0.07 |
| S28 | 20.11 | 21.78 | 0.34 | 21.96 | 0.05 | 22.68 | 0.10 | 22.93 | 0.07 | 20.83 | 22.37 | 0.06 | 23.48 | 0.03 | 22.40 | 0.07 | 23.61 | 0.09 |
| S29 | 33.38 | 35.44 | 0.26 | 33.92 | 0.06 | 32.85 | 0.16 | 32.63 | 0.09 | 35.29 | 37.27 | 0.82 | 37.10 | 0.37 | 34.58 | 0.31 | 33.79 | 0.17 |
| S30 | 19.97 | 21.53 | 0.08 | 22.36 | 0.30 | 22.96 | 0.16 | 23.92 | 0.06 | 20.43 | 21.63 | 0.04 | 22.83 | 0.03 | 23.80 | 0.05 | 24.86 | 0.07 |
| S31 | 30.59 | 30.71 | 0.13 | 31.72 | 0.23 | 29.85 | 0.38 | 32.82 | 0.28 | 30.99 | 32.11 | 0.17 | 33.10 | 0.19 | 32.22 | 0.03 | 34.19 | 0.35 |
| S32 | 13.98 | 15.67 | 0.15 | 16.30 | 0.04 | 15.17 | 0.09 | 15.42 | 0.12 | 13.83 | 15.54 | 0.05 | 17.08 | 0.04 | 14.62 | 0.03 | 16.03 | 0.05 |
| S33 | 22.60 | 23.10 | 0.08 | 23.69 | 0.28 | 25.88 | 0.12 | 26.34 | 0.09 | 23.35 | 23.61 | 0.11 | 23.62 | 0.04 | 25.74 | 0.13 | 26.66 | 0.07 |
| S34 | 32.03 | 35.16 | 0.37 | 35.09 | 0.84 | 38.80 | 0.58 | ND | ND | 33.10 | 35.08 | 0.52 | 40.38 | 0.23 | 34.72 | 0.00 | ND | ND |
| S35 | 29.44 | 36.83 | 0.00 | 36.87 | 0.00 | 36.55 | 0.00 | ND | ND | 28.49 | 36.32 | 0.00 | ND | ND | ND | ND | ND | ND |
| S36 | 34.93 | 34.35 | 0.81 | 37.14 | 0.00 | ND | ND | ND | ND | 35.69 | 37.04 | 0.88 | 39.86 | 0.03 | ND | ND | ND | ND |
| S37 | 36.82 | 34.21 | 0.17 | 36.56 | 0.00 | 36.97 | 0.00 | 36.22 | 0.00 | 35.87 | 35.57 | 1.02 | 41.71 | 0.00 | ND | ND | ND | ND |
| S38 | 32.04 | 33.33 | 0.55 | 33.08 | 0.19 | 36.32 | 0.73 | 35.58 | 1.07 | 32.04 | 33.20 | 0.49 | 33.10 | 0.30 | 34.54 | 0.48 | 39.13 | 0.21 |

**Table S2.** Pool performed after RNA extraction. Each mean sample represents one patient tested. Standard deviation (SD) represents the technical replicates.

| **Sample** | **N1** | | | | | | | | | **N2** | | | | | | | | | | |
| --- | --- | --- | --- | --- | --- | --- | --- | --- | --- | --- | --- | --- | --- | --- | --- | --- | --- | --- | --- | --- |
|  | **Absolute CT** | **Pool 1:4** | | **Pool 1:8** | | **Pool 1:16** | | **Pool 1:32** | | **Absolute CT** | **Pool 1:4** | | **Pool 1:8** | | | **Pool 1:16** | | | **Pool 1:32** | |
|  |  | **Mean** | **SD** | **Mean** | **SD** | **Mean** | **SD** | **Mean** | **SD** |  | **Mean** | **SD** | **Mean** | **SD** | **Mean** | | **SD** | **Mean** | | **SD** |
| R1 | 28.23 | 27.31 | 0.20 | 28.36 | 0.25 | 32.14 | 0.81 | - | - | - | 28.31 | 0.12 | 29.99 | 0.19 | 29.88 | | 0.04 | - | | - |
| R2 | 29.73 | 33.41 | 0.33 | 34.34 | 0.49 | 36.22 | 0.58 | - | - | - | 34.14 | 0.30 | 36.94 | 1.16 | 36.69 | | 0.39 | - | | - |
| R3 | 32.20 | 32.56 | 0.59 | 33.27 | 0.34 | 33.73 | 0.20 | - | - | - | 32.68 | 0.07 | 35.08 | 0.44 | 35.07 | | 0.09 | - | | - |
| R4 | 29.78 | 31.10 | 0.45 | 32.55 | 0.43 | 33.44 | 0.40 | - | - | - | 32.39 | 0.43 | 33.10 | 0.34 | 34.24 | | 0.48 | - | | - |
| R5 | 30.58 | 32.99 | 0.85 | 34.95 | 0.47 | 36.16 | 0.47 | - | - | - | 34.05 | 0.36 | 35.43 | 0.38 | 34.22 | | 0.38 | - | | - |
| R6 | 26.88 | 28.75 | 0.05 | 29.75 | 0.08 | 30.10 | 0.10 | - | - | - | 28.60 | 0.28 | 29.27 | 0.25 | 29.32 | | 0.10 | - | | - |
| R7 | 21.47 | 20.91 | 0.07 | 22.23 | 0.08 | 24.89 | 0.09 | - | - | - | 20.79 | 0.10 | 21.58 | 0.11 | 24.32 | | 0.10 | - | | - |
| R8 | 31.37 | 31.45 | 0.17 | 33.02 | 0.41 | 34.46 | 0.70 | - | - | - | 34.03 | 0.41 | 36.24 | 0.36 | 40.43 | | 0.00 | - | | - |
| R9 | 31.87 | 30.59 | 0.08 | 31.81 | 0.46 | 33.32 | 0.21 | - | - | - | 32.43 | 0.30 | 34.14 | 0.40 | 35.11 | | 0.44 | - | | - |
| R10 | 35.33 | 33.02 | 0.16 | 34.95 | 0.81 | 34.61 | 0.18 | - | - | - | 35.37 | 0.47 | 37.82 | 0.76 | 35.82 | | 0.31 | - | | - |
| R11 | 34.32 | 32.85 | 0.42 | 34.02 | 0.69 | 34.06 | 0.42 | - | - | - | 35.14 | 0.39 | 35.41 | 0.22 | 35.52 | | 4.23 | - | | - |
| R12 | 34.95 | 33.64 | 0.55 | 34.73 | 0.12 | 34.80 | 0.12 | - | - | - | 35.73 | 0.34 | 35.86 | 0.04 | 36.40 | | 0.04 | - | | - |
| R13 | 36.29 | 34.78 | 0.27 | 35.02 | 0.71 | 36.04 | 0.15 | - | - | - | 36.26 | 0.18 | 39.96 | 0.45 |  | |  | - | | - |
| R14 | 22.93 | 25.14 | 0.07 | 26.43 | 0.04 | 27.42 | 0.11 | 27.37 | 0.15 | 24.07 | 25.79 | 0.01 | 27.25 | 0.05 | 29.59 | | 0.49 | 29.93 | | 0.56 |
| R15 | 15.29 | 15.44 | 0.07 | 16.88 | 0.10 | 17.77 | 0.05 | 20.33 | 0.05 | 15.00 | 14.80 | 0.10 | 15.91 | 0.01 | 17.31 | | 0.06 | 20.01 | | 0.06 |
| R16 | 15.50 | 17.49 | 0.03 | 18.50 | 0.10 | 19.64 | 0.06 | 20.46 | 0.08 | 15.57 | 18.83 | 0.04 | 17.34 | 0.06 | 19.86 | | 0.04 | 20.39 | | 0.06 |
| R17 | 18.59 | 19.75 | 0.05 | 20.60 | 0.06 | 21.00 | 0.04 | 23.34 | 0.07 | 19.00 | 19.99 | 0.11 | 21.19 | 0.01 | 21.38 | | 0.03 | 24.05 | | 0.06 |
| R18 | 17.74 | 20.02 | 0.04 | 21.27 | 0.01 | 22.12 | 0.15 | 22.44 | 0.06 | 18.15 | 22.03 | 0.05 | 23.10 | 0.05 | 24.43 | | 0.15 | 25.79 | | 0.10 |
| R19 | 33.38 | 36.53 | 1.10 | 36.90 | 0.43 | 37.00 | 0.21 | 37.11 | 0.14 | 35.29 | 37.99 | 0.95 | 39.03 | 0.49 | 38.89 | | 0.00 | 39.60 | | 0.00 |
| R20 | 19.97 | 22.95 | 0.03 | 23.97 | 0.03 | 25.03 | 0.05 | 25.98 | 0.11 | 20.43 | 22.97 | 0.02 | 24.02 | 0.05 | 25.07 | | 0.04 | 26.00 | | 0.03 |
| R21 | 30.59 | 32.47 | 0.14 | 33.49 | 0.51 | 34.04 | 0.51 | 35.39 | 0.00 | 30.99 | 33.56 | 0.19 | 34.26 | 0.13 | 35.10 | | 0.12 | 36.44 | | 0.29 |
| R22 | 13.98 | 16.24 | 0.05 | 17.38 | 0.07 | 18.53 | 0.01 | 19.23 | 0.00 | 13.83 | 15.88 | 0.01 | 17.06 | 0.02 | 17.97 | | 0.04 | 19.22 | | 0.03 |
| R23 | 22.60 | 25.06 | 0.00 | 26.33 | 0.08 | 27.59 | 0.09 | 28.33 | 0.05 | 23.35 | 25.32 | 0.06 | 26.49 | 0.09 | 27.58 | | 0.11 | 28.53 | | 0.08 |
| R24 | 32.04 | 34.97 | 0.83 | 34.46 | 0.63 | 35.46 | 0.86 | 35.75 | 0.59 | 32.04 | 36.70 | 0.23 | 38.45 | 1.00 | 41.03 | | 0.00 | 39.09 | | 0.08 |

(-) represents spots not evaluated.

**Table S3.** Ct mean and standard deviation (SD) of RT-qPCR analysis performed with clinical samples using singleplex, multiplex and multiplex plus pooling strategies.


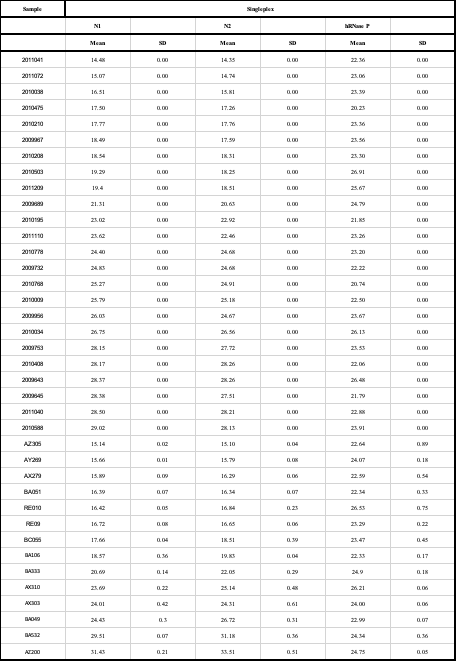


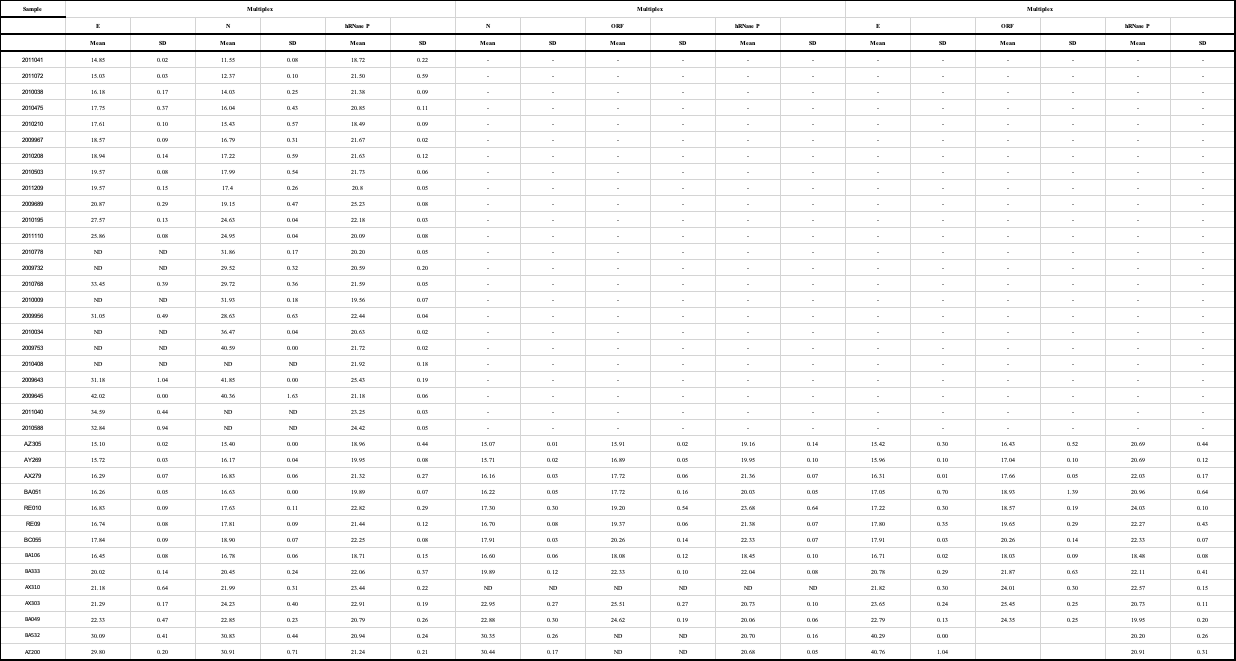


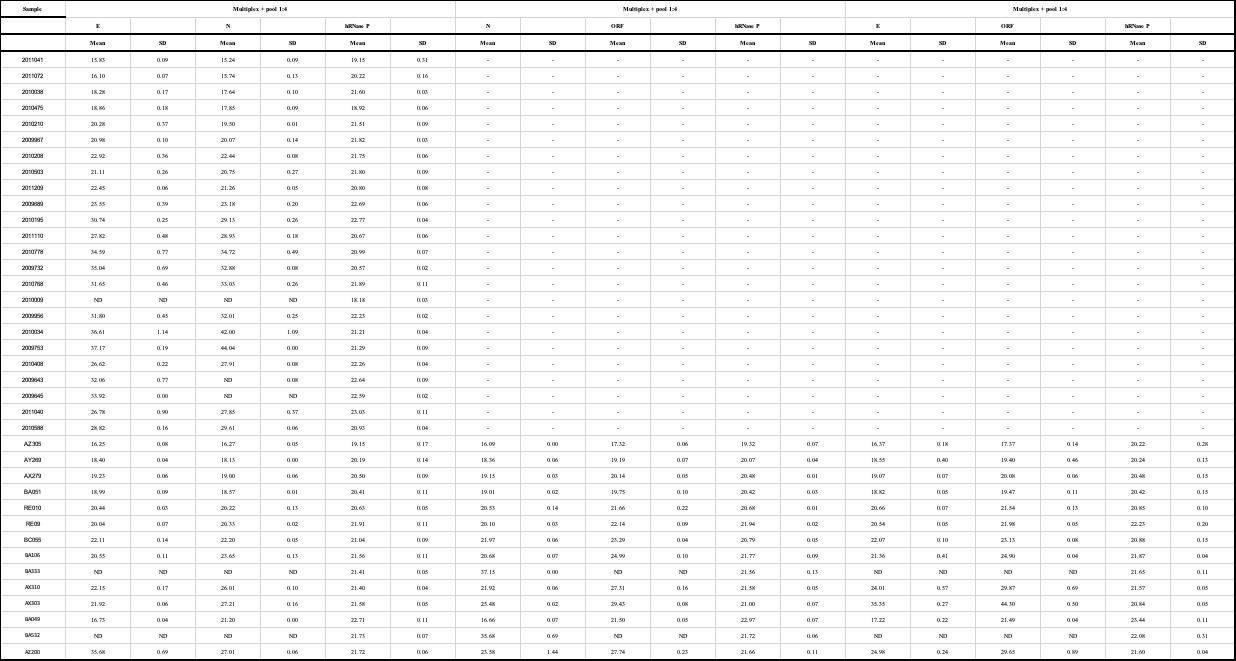


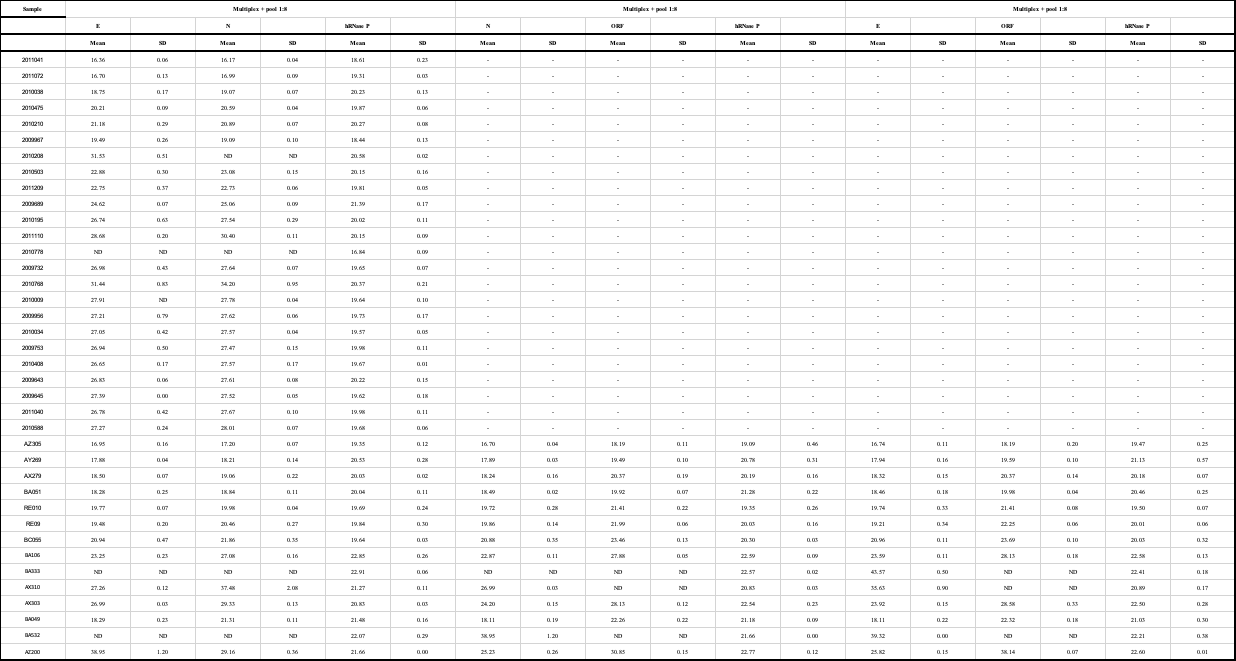


(-) represents spots not evaluated.
